# Using a Second Order Sigma-Delta Control to Improve the Performance of Metal-Oxide Gas Sensors

**DOI:** 10.3390/s18020654

**Published:** 2018-02-23

**Authors:** Lukasz Kowalski, Joan Pons-Nin, Eric Navarrete, Eduard Llobet, Manuel Domínguez-Pumar

**Affiliations:** 1 Micro and Nano Technologies Group, Electronic Engineering Department, Universitat Politècnica de Catalunya—BarcelonaTech, 08034 Barcelona, Spain; lukasz.kowalski@upc.edu (L.K.); joan.pons@upc.edu (J.P.-N.); 2 MINOS-EMaS Group, Electronic Engineering Department, Universitat Rovira i Virgili, 43003 Tarragona, Spain; eric.navarrete@urv.cat (E.N.); eduard.llobet@urv.cat (E.L.)

**Keywords:** sigma-delta modulation, metal-oxide sensors, gas sensors, electrochemical impedance

## Abstract

Controls of surface potential have been proposed to accelerate the time response of MOX gas sensors. These controls use temperature modulations and a feedback loop based on first-order sigma-delta modulators to keep constant the surface potential. Changes in the surrounding gases, therefore, must be compensated by average temperature produced by the control loop, which is the new output signal. The purpose of this paper is to present a second order sigma-delta control of the surface potential for gas sensors. With this new control strategy, it is possible to obtain a second order zero of the quantization noise in the output signal. This provides a less noisy control of the surface potential, while at the same time some undesired effects of first order modulators, such as the presence of plateaus, are avoided. Experiments proving these performance improvements are presented using a gas sensor made of tungsten oxide nanowires. Plateau avoidance and second order noise shaping is shown with ethanol measurements.

## 1. Introduction

Interest in metal-oxide (MOX) gas sensors has grown significantly during the recent years. Different materials, such as SnO_2_, WO_3_, or ZnO, and specific fabrication techniques have been developed to form the sensing layers of such sensors, often structured as nanoneedles, nanotubes, nanorods, etc. The high surface-to-volume ratios of these nanostructures allow high levels of interaction with the environment, thus providing high sensitivities. Good stability, reduced cost, low power consumption, and compatibility with semiconductor fabrication processes are other advantages of this type of sensors [1,2,3,4,5]. All this makes them excellent candidates in applications such as detection of hazardous gases, pollution observation, or detection of gas leaks [6,7,8]. 

The mode of operation of the MOX gas sensors usually consists of monitoring the conductivity of the sensing layer. Since this layer is a semiconductor, its conductance strongly depends not only on the temperature but also on the chemical reactions involved in the gas adsorption and ionization processes [9]. Moreover, the conductivity of the sensor layer can be seen as the result of two simultaneous competing mechanisms that have different time scales. The first mechanism is due to the temperature applied, which produces redistribution in the energies of the charge carriers and causes fast changes in the conductivity of the layer; the second mechanism consists in changes in the chemical reaction rates with the gas species, which generate slow changes in the conductivity of the sensing layer. MOX sensors are usually operated in open-loop at constant-high temperature, of 100 °C and above, depending on the specific materials of the sensing layer. To reach and maintain constant operating temperature, heaters are usually embedded in the sensors.

However, the performance of MOX-based gas sensors becomes limited by their slow time response to changes in gas concentration and, in some cases, by unwanted long term drifts. As a first step towards studying these issues, dynamic models of the sensors have been proposed [10,11], and it is widely accepted that nonlinear models must be used to describe the evolution with time of the chemical reactions in the layer. As a consequence, complex digital-processing tools, such as neural networks [12], probabilistic state estimation [13], reservoir computing [14], and support vector machines [15] are used to improve sensor performance. Temperature modulations have also been used in works reported in the literature with MOX gas sensors to reduce measurement uncertainty [13], to improve feature extraction [16,17,18,19], or to reduce power consumption [20,21]. Additionally, single walled carbon nanotubes have been used in conjunction with MOX modulated in temperature to reduce power consumption [22].

A new approach for smart operation of MOX gas sensors has been proposed recently by the authors [23]. There, a closed-loop technique, inspired in first order sigma-delta modulation, is applied to enforce a sliding mode control on the state variables of the sensor [24]. In particular, the feedback loop produces the temperature modulations necessary to operate the sensor under constant surface potential. In this case, the output of the sensor is the average temperature applied to the sensing layer. This strategy allows the time dynamics of the system to be changed: since the control variable (the surface potential) is constant, the excursion of one or several state variables is reduced and the time response of the system no longer depends on its own free (and slow) dynamics, but on the marginal dynamics obtained within the control surface. This way, fast time responses with a MOX gas sensor have been demonstrated [23]. 

However, some known issues of first-order sigma-delta modulators can limit the effectiveness of the technique proposed in [23]. One is the presence of a Devil’s Staircase fractal, a typical effect when leaky integrators are used [25]; a fractal plateau is in practice a “dead zone” that cannot be observed or controlled, thus hindering the possibility of having a good control in certain cases. Another issue is poor quantization noise shaping, which can pose a problem for retrieving real-time information about the sensing layer. According to this, this paper introduces a new second-order sigma-delta strategy to control the chemical resistance of MOX gas sensors. This method improves the one previously proposed, providing second order quantization noise shaping, smoother sensor responses, and allows for the avoidance of the plateaus observed in the first-order approach. The feasibility and the features of the new method are demonstrated experimentally through extensive comparisons of both control methods in gas sensing applications with tungsten oxide nanowires.

## 2. Materials and Methods

### 2.1. First and Second Order Sigma-Delta Loops for Gas Sensing

As it has been mentioned in the Introduction, the typical operation of chemical gas sensors based in semiconductor metallic oxide (MOX) layers consists in keeping such sensing layers at constant temperature and monitor changes in its resistivity. Under this approach, the time response of the sensor is completely determined by the dynamics of the surface adsorption and ionization reactions, which can be generally very slow. 

In order to improve the time response of the sensors, the control proposed in [23] operates the sensor under a new condition: Constant Surface Potential Operation. This is done by implementing a sliding mode controller [26] using the scheme of a first order sigma-delta modulator [24]. Sliding mode controllers confine the dynamics of the dynamical system to a predetermined control surface. By doing this, the dynamics of the whole system can be completely changed under some conditions. This control can be performed using the sigma-delta approach, in which the topology of these analog-to-digital converters is used to obtain the desired sliding motion on the control surface. In our case, then the control surface is constant Surface Potential. 

Now, the conductivity of the chemical sensing layer follows this general expression [23]:

(1) $$G=G_{0}\left(T\right)\exp\left(-\frac{qV_{s}}{kT}\right),$$

in which $G_{0}\left(T\right)$ is a factor depending on temperature, *T*, *q* is the electron-elementary charge, $V_{s}$ is the surface potential, and *k* is the Boltzmann constant. From this expression it is clear that in order to keep $V_{s}$ constant, the conductivity of the sensing layer must be kept constant and measured at constant temperature. 

**Figure 1:**
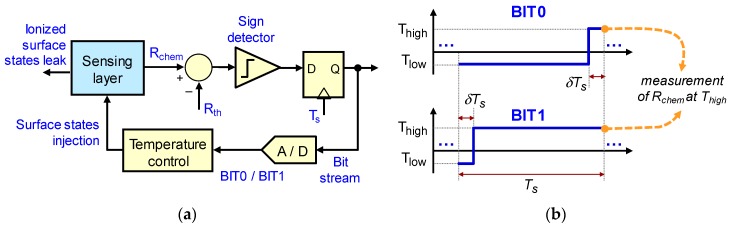
(**a**) First-order sigma-delta modulator topology to control the chemical resistance of the metal-oxide sensing layer. At each sampling time *T_S_*, depending on whether the chemical resistance *R_chem_*, measured at the reference temperature *T_high_*, is below (or above) the desired value *R_th_*, a BIT1 (or BIT0) temperature waveform is applied to the sensor during the next sampling period; (**b**) parameters of the BIT0 and BIT1 waveforms.

The control proposed in [23] is shown in Figure 1a. It achieved the constant SP operation by applying an adequate sequence of temperature waveforms to the sensor. These waveforms, called BIT0 and BIT1, can be seen in Figure 1b. By periodically sampling the conductivity of the sensing layer, at the end of each sampling period, it is possible to monitor changes in *Vs*, since both waveforms end with the same temperature value *T_high_*. The control is designed to apply, for the following sampling period $\left[nT_{s},\left(n+1\right)T_{s}\right)]$, a BIT1 waveform if $G\left[nT_{s}\right]>G_{target}$ or a BIT0 if $G\left[nT_{s}\right]<G_{target}$. This way, it is possible to change the average temperature in the sensor, while making decisions based on the conductivity of the sensing layer, measured at the same temperature. 

This control replicates the usual circuit topology of a 1st order sigma-delta modulator. The sensing layer can be seen as a reservoir of ionized surface states, which can be negatively or positively charged. In an oxidizing atmosphere, increasing (decreasing) the average temperature will increase (decrease) the adsorption of gas molecules that, when ionized, will increase (decrease) the total negative ionized surface states. In this case, the average temperature generated by the control must be able to keep constant the interchange of ionized surface states with the surrounding atmosphere by applying a suitable sequence of temperature waveforms. Changes in the atmosphere are therefore compensated for by changes in the average temperature generated by the control.

**Figure 2:**
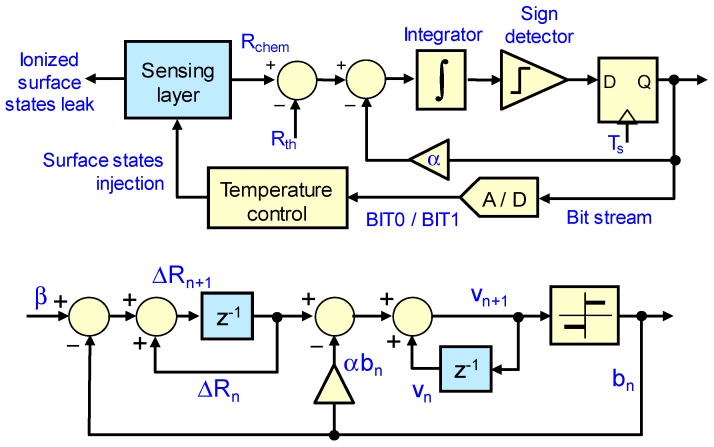
Block diagram (**Top**) and equivalent sampled circuit (**Bottom**) of the 2nd order sigma-delta topology designed to control the chemical resistance of the MOX sensing layer.

As it has been mentioned before, this paper presents a second order sigma-delta topology for surface potential control in MOX-based gas sensors. By adding an integrator to the control loop, it is possible to obtain a second order zero in the quantization noise at zero frequency [27]. This integrator is implemented numerically, see Figure 2, and good values of the α parameter are empirically found. Besides the improvement in the quantization noise, second order modulators do not present plateaus in the case of leaky integrators. On the other hand, they can become unstable under some conditions [27]. 

Figure 2 presents the qualitative translation of the 2nd order control loop to the standard sigma-delta idealized representation. Parameter *β* represents the continuous leak of ionized surface states, $\Delta R_{n}$ represents the differences at the sampling times $nT_{s}$ between the resistivity of the layer and the target value set $R_{chem}\left(nT_{s}\right)-R_{th}$, and $v_{n}$ are the values of the second numerical integrator. The functions of the first integrator are performed by the sensing layer itself, seen as a reservoir of ionized surface states. The purpose of the control circuit is therefore to cancel the value at the output of this second integrator by applying an adequate sequence of BIT0/BIT1 waveforms to the sensor.

### 2.2. Description of the Gas Sensors

#### 2.2.1. Sensing Layer Synthesis

The sensing layers consist of pristine tungsten oxide nanowires directly grown on top of the membranes of a 4-element micro-machined silicon transducer employing an Aerosol Assisted Chemical Vapor Deposition (AACVD) process. Each membrane within the 4-element chip contains POCl_3_-doped polysilicon heaters (16 Ω/sq, 0.47 μm thickness, and TCR = 6.79 × 10^−4^/°C) and platinum electrodes (0.2 μm thickness, electrode gap = 100 μm). To electrically insulate the electrodes on top from the heater, 800-nm-thick silicon oxide layers were deposited by Low Pressure Chemical Vapor Deposition (LPCVD). In the AACVD growth of tungsten oxide nanowires, tungsten hexacarbonyl (50 mg, Sigma-Aldrich, Saint Louis, MO, USA, ≥97%) dissolved in a mixture of acetone and methanol (15 mL of acetone and 5 mL of methanol, Sigma-Aldrich, ≥99.6%) was used. The solution was kept in a flask and placed in an ultrasonic humidifier. 

**Figure 3:**
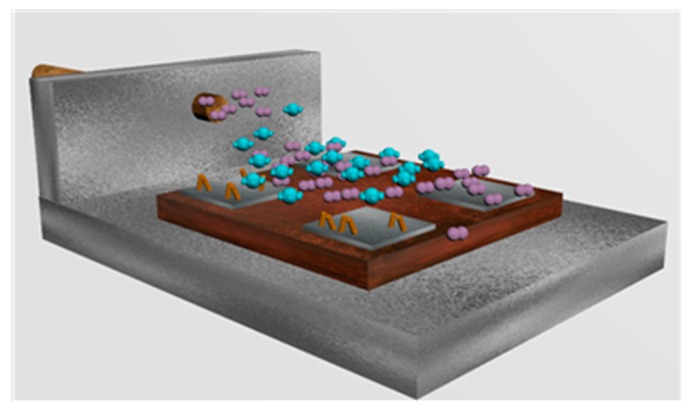
Artistic view inside of the hot wall reactor during the AACVD process. A nitrogen flow carries the aerosol droplets of solvent containing the organic precursor.

The resulting aerosol was transported to the reactor by a 500 mL/min flow of nitrogen. The substrates were placed inside the reactor and the whole system was heated up to 370 °C (see Figure 3). A mask was placed on top of the substrate to protect the contact pads of the heater and electrodes, leaving exposed the electrode areas only. The reactor outlet was vented directly into the extraction system of a fume cupboard. The deposition time ranged between 30 to 40 min, until all of the precursor had passed through the reactor. At the end of the growth, the flow of nitrogen was interrupted, and the substrates were kept in the reactor at 370 °C for another 60 min. This helps the removal of the precursor residues and further oxidizes nanowires. Films have a pale-yellow color, which indicates that a close to stoichiometry tungsten oxide is obtained. Finally, the sensors were wire-bonded to standard TO-8 packages. 

#### 2.2.2. Material Characterization

**Figure 4:**
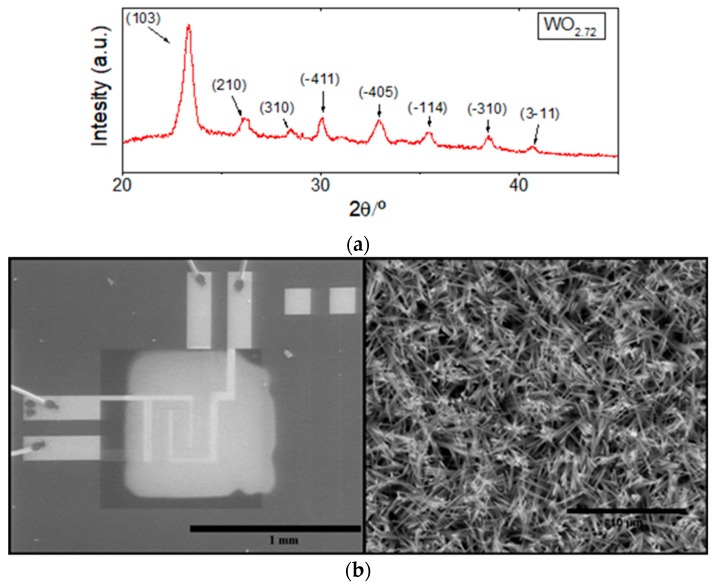
(**a**) XRD results obtained for typical tungsten oxide nanowire films. Tungsten oxide is single crystalline and belongs to the monoclinic phase; (**b**) low magnification micrograph showing the AACVD grown film on top of the electrode area of a sensor within the 4-element transducer (**Left**). Higher magnification micrograph showing the typical microstructure of the AACVD grown tungsten nanowire films (**Right**).

The morphology and crystalline phase of the gas sensitive films were analyzed using an Environmental Scanning Electron Microscope (ESEM) and X-Ray Diffraction (XRD). Figure 4a shows typical XRD results for the films grown. These results indicate that a slightly oxygen-defective tungsten oxide is obtained, which corresponds to WO_2.72_ nanowires that have a P2/m belonging to the monoclinic system, in accordance to the JPCD card no. 73-2177. Figure 4b shows ESEM results. The lower magnification micrograph shows one membrane with interdigitated electrodes and, in light grey, the sensing layer composed of WO_3_ nanowires. The higher magnification micrograph shows a closer view of the nanowire film.

### 2.3. Experimental Setup

The experiments designed for this work aimed to compare the effectiveness and performance of the first and second order control methods discussed above; they included measurements with the tungsten oxide nanowire sensors that are presented, both in controlled atmospheres of synthetic air and of synthetic air with small concentrations of ethanol. 

**Figure 5:**
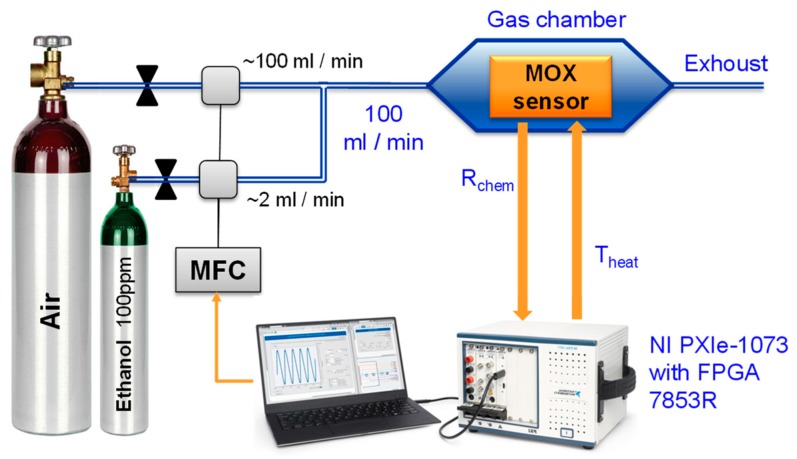
Description of the experimental setup.

Accordingly, the measurement setup described in Figure 5 has been used. The sensor was placed inside a gas chamber. To set different target gas concentrations, a calibrated ethanol cylinder with air as balance gas was used. This was further diluted by employing a cylinder of dry air and a computer-controlled mass flow meter system. The total flow into the chamber was kept constant at 100 mL/min throughout the experiments. The dead volume within the gas chamber was 4 mL. The periodical measurements of the chemical resistance of the sensing layer and the application of the BIT0 and BIT1 temperature waveforms are implemented using a standard FPGA-based National Instruments PXIe-1073 acquisition equipment controlled from the same computer. The experimental data was post-processed using standard MatLab software. 

**Figure 6:**
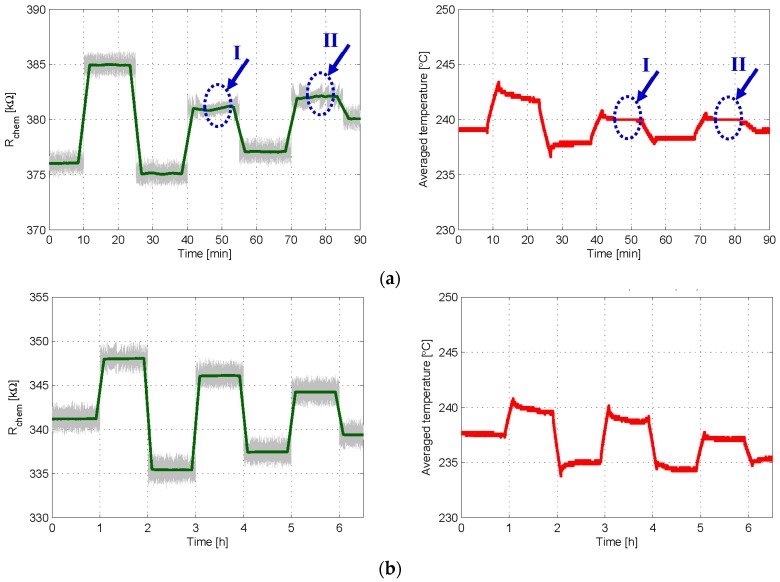
Experimental results in which 1st and 2nd order controls are used to obtain a given sequence of target chemical resistances *R_th_*. (**a**) Time evolution of the chemical resistance (**Left**) and of the average temperature provided by the 1st order loop (**Right**); *R_th_* was set to 376, 385, 375, 381, 377, 382, and 380 kΩ in 15 min intervals; *T_high_* = 280 °C, *T_low_* = 200 °C, δ = 25%, and *T_S_* = 1 s; (**b**) same results when 2nd order control was applied to set R_chem_ to 341, 348, 335, 346, 337, 344, and 339 kΩ in 60 min intervals; *T_high_* = 280 °C, *T_low_* = 200 °C, α = 2 kΩ, δ = 20% and *T_S_* = 2 s. In left plots, the grey lines are the raw signals at the sampling frequency, while the green one is the moving average obtained with 200 samples.

In each experiment reported in this paper, a previous characterization of the sensor layer with the temperature was performed to choose the appropriate values of the BIT0 and BIT1 and of the control parameters: *T_high_*, *T_low_*, *R_th_*, etc. Concretely, the same procedure as in [23] was used, e.g., see details in Figure 6 and related text of this reference.

## 3. Results and Discussion

In accordance with the theoretical expectations discussed above, the objective of this section is to demonstrate experimentally that, under certain conditions, the 1st order loop control may suffer from “plateau-related” phenomena, which in practice leads to transient losses of control of the chemical resistance, and that using a 2nd order loop allows for the avoidance this problem. This section also intends to verify the improvement of the quantization noise shaping when higher-order loops are used. All these effects are further investigated using an ethanol gas-sensing application as reference. 

### 3.1. Experiment Set 1—Chemical Resistance Control with First and Second Order Sigma-Delta Loops

The first set of experiments aimed to investigate the feasibility of both control loops to obtain a sequence of arbitrary values of chemical resistance. Accordingly, in the experiment reported in Figure 6a, the sensor was placed in synthetic air, and the 1st order control loop (see Figure 1) with a sampling time *T_S_* = 1 s was used to set seven different values of *R_th_* in 15 min intervals. It is seen in Figure 6a that the 1st order control works mostly fine, since the target chemical resistances are successfully achieved and the bit stream average, or the temperature average applied to the sensor, tends to stabilize after each *R_th_* step. For example, at *t* = 10 min *R_chem_* must be increased, and therefore the control loop applies more BIT1s (*T_high_* dominant), increasing the oxygen adsorption in the sensing layer, until *R_chem_* = *R_th_* is reached; from then on, the bit stream/temperature average slowly tends to the value necessary to keep *R_chem_* constant. However, two “plateau events”, labeled as I and II in Figure 6a, are also observed. During these events, the average temperature injected into the sensor becomes locked to 240 °C (which corresponds to the same average number of BIT1s and BIT0s, i.e., *b_n_* = 0.5 or the temperature value (*T_high_* + *T_low_*)/2), and, in practice, the system behaves as in open-loop, thus losing control on the value of *R_chem_*.

On the other hand, Figure 6b shows the result of an experiment similar to that of Figure 6a, using the 2nd order controller (see Figure 2) with *T_S_* = 2 s. The curves of Figure 6b demonstrate that the 2nd order loop allows for the successful achievement of all target values of *R_chem_* and that plateau-related events are no longer seen, even using a slower sampling rate.

**Figure 7:**
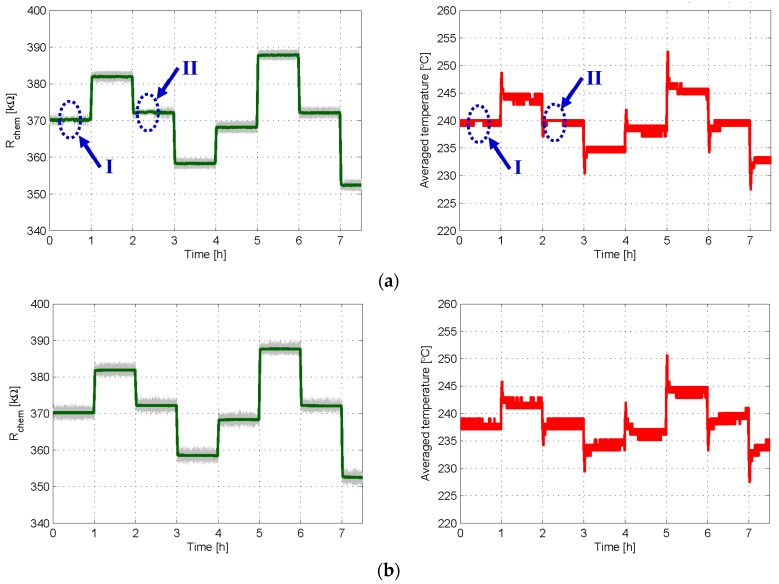
Experimental results in which 1st and 2nd order controls are used to obtain the same sequence of target chemical resistances *R_th_* = 370, 382, 372, 358, 368, 388, 372, and 352 kΩ in 60 min intervals. In both cases, *T_high_* = 280 °C, *T_low_* = 200 °C, δ = 20%, and *T_S_* = 2 s; α = 2 kΩ in the 2nd order case; (**a**) time evolution of the chemical resistance (**Left**) and of the average temperature provided by the 1st order loop (**Right**); (**b**) same results when 2nd order control was applied. In left plots, the grey lines are the raw signals at the sampling frequency, while the green one is the moving average obtained with 50 samples.

In the experiment reported in Figure 7, the sensor is again placed in synthetic air and both control loops are used to obtain the same sequence of target chemical resistances, with *T_S_* = 0.5 s. According to sigma-delta theory, in 1st order loops with leaky integrators the presence of plateaus becomes less evident for increasing values of the sampling frequency 1/*T_S_* [25]. Note that, although the sampling frequency doubles that of Figure 6a, plateau-related events are still observed with 1st order control, see Figure 7a. On the other hand, no plateaus are seen in the 2nd order case, see Figure 7b. 

This means that even by doubling the sampling frequency of the experiment in Figure 6, first order controls may still produce plateaus (Figure 7). However, using the 2nd order control method allows for the avoidance of these plateau-related events, thereby improving the performance of the sensor as a system. 

**Figure 8:**
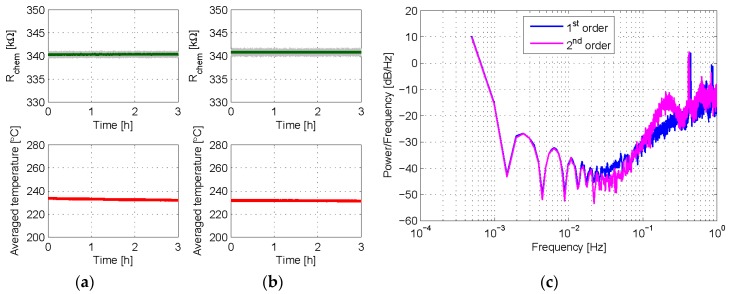
Experimental results in which 1st and 2nd order controls are applied to set *R_chem_* to 340 kΩ for 3 h. In both cases, *T_high_* = 290 °C, *T_low_* = 200 °C, δ = 25%, and *T_S_* = 0.5 s; α = 1.4 kΩ in the 2nd order case. (**a**) Chemical resistance of the sensing layer (**Top**) and averaged temperature provided by the 1st order loop (**Bottom**); (**b**) same results as provided by the 2nd order loop; (**c**) power spectrum densities after 16,384 samples of the bit streams, obtained with standard P-Welch MatLab estimation. In top (**a**,**b**) plots, the grey lines are the raw signals at the sampling frequency, while the green one is the moving average obtained with 200 samples.

The last experiment reported in this section aims to compare the behavior of the two control loops in terms of quantization noise in the output bit stream. As it has been said before, a first order sigma-delta topology produces a zero of first order. This means a slope of approximately 20 dB/decade in the quantization noise near zero frequency. With the second order control, a slope of approximately 40 dB/decade should be obtained. To observe this effect, the sensor was placed again in synthetic air and each type of control with *T_S_* = 0.5 s was applied for 3 h to set a target chemical resistance of 340 kΩ. As shown in Figure 8a,b, constant chemical resistance and constant average temperature are successfully achieved with both control methods. 

The spectral power density corresponding to 16,384 samples of stabilized bit stream was calculated in each case. Figure 8c compares the obtained spectra. It is seen there how the presence of the additional integrator in the 2nd order controller produces noticeable differences. With the 2nd order control, the response at low frequencies becomes improved, and the quantization noise is rolled out of the band of interest with a slope of 40 dB/decade, being this figure 20 dB/decade in the 1st order case. Let us note that these results strongly resemble those obtained in other works, in which 1st and 2nd order sigma-delta loops are applied to thermal modulators for flow sensing applications [28] and to control dielectric charging in electrostatic MEMS [29] and CMOS capacitors [30]. 

### 3.2. Experiment Set 2—Gas Sensing with First and Second Order Sigma-Delta Loops

This section investigates the improvements introduced by the 2nd order control in the performance of the sensor. To this effect, different ethanol concentrations have been applied, while a control loop is being used to set the condition “constant chemical resistance *R_chem_* measured at constant temperature *T_high_*” in the sensing layer.

**Figure 9:**
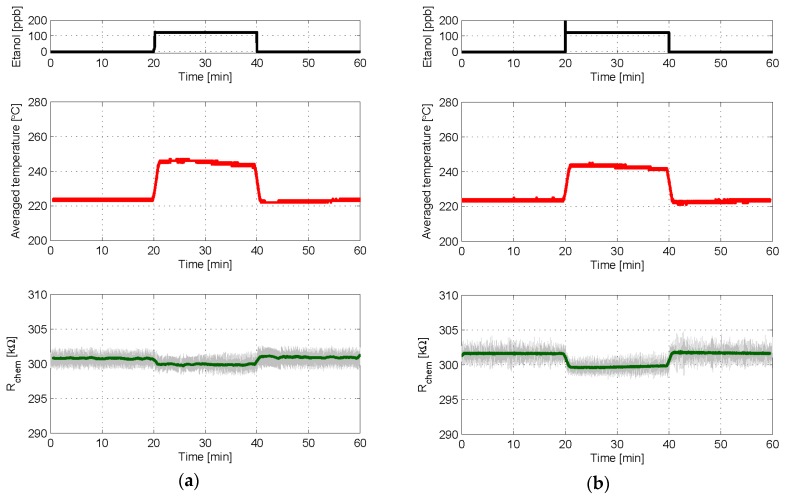
Experimental results in which either a 1st or a 2nd order control loop is used to set *R_chem_* to 300 kΩ while a step of ethanol concentration was applied to the sensor. *T_high_* = 290 °C, *T_low_* = 160 °C, δ = 25%, and T_S_ = 1 s in both cases; α = 3 kΩ in the 2nd order case. (**a**) Evolution with time of the ethanol concentration (**Top**), the average temperature provided by the control loop (**Middle**), and the chemical resistance (**Bottom**) when 1st order control was used; (**b**) same results for 2nd order control. In bottom plots the grey lines are the raw signals at the sampling frequency, while the green one is the moving average obtained with 60 samples.

In the first two experiments, the sensor was initially under synthetic air for 20 min, then a concentration of 125 ppb of ethanol was applied for another 20 min, and finally synthetic air was applied again for 20 min. Either a 1st or a 2nd order control loop was applied on each experiment, with the same BIT parameters, to set the chemical resistance to 300 kΩ. A result comparison of these experiments is available in Figure 9. It is seen there that, with both controls, the ethanol step produces a positive step of the average temperature applied, which is the sensor output. This is consistent with the fact that ethanol is a reducing gas, but its mixing with air makes the environment remain still oxidant, and therefore it is necessary to increase the ratio of BIT1s (*T_high_* dominant or temperature increase) to keep almost constant the chemical resistance. These results indicate that the sensor response under both controls is rather similar and, in particular, that the 2nd order controller does not reduce its speed, a key feature of this kind of sensors. Additionally, due to the improved response against quantization noise provided by the integrator, a noticeably smoother control of the chemical resistance is achieved when using the 2nd order loop.

**Figure 10:**
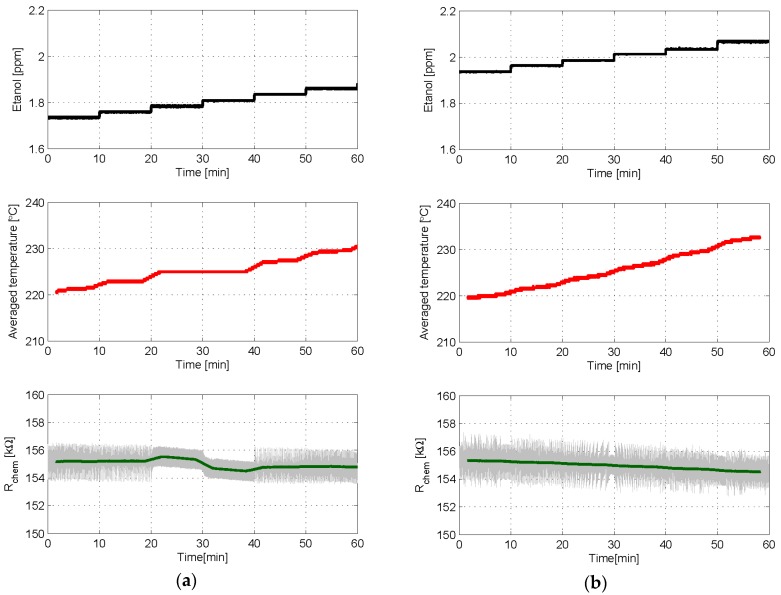
Experimental results in which either a 1st or a 2nd order control loop has been used to set *R_chem_* to 155 kΩ when a sequence of small steps of ethanol of 25 ppb concentration was applied to the sensor in 10 min intervals. *T_high_* = 290 °C, *T_low_* = 160 °C, δ = 25%, and *T_S_* = 1 s in both cases; α = 3 kΩ in the 2nd order case. (**a**) Evolution with time of the ethanol concentration (**Top**), the averaged bit stream provided by the control loop (**Middle**), and the chemical resistance (**Bottom**) when 1st order control was used; (**b**) same results when 2nd order control was used. In bottom plots the grey lines are the raw signals at the sampling frequency, while the green one is the moving average obtained with 200 samples.

The last two experiments reported consist in applying a sequence of small-increasing steps of ethanol concentration to the sensor, while either a 1st or a 2nd order control loop is configured to set the chemical resistance of the sensing layer to 155 kΩ. Each step increases the ethanol concentration in 25 ppb and lasts for 10 min. The same set of BIT0 and BIT1 parameters was used in both controls, and the results are shown in Figure 10. The unwanted effect of a plateau is clearly seen in the case of the 1st order control, Figure 10a. Specifically, the sensor becomes locked to a constant average temperature (again corresponding to (*T_high_ + T_low_*)/*2*, *b_n_* = 0.5), thus producing the same output value, for two different steps of gas concentration. 

This insensitivity to changes in gas concentration does not exist in the case of the 2nd order control, which provides an output behavior that clearly follows the increasing-step shape of ethanol concentration applied, see Figure 10b. We can conclude that the 2nd order controller ensures a 1-to-1 relationship between gas excitation and sensor response.

## 4. Conclusions

A new second-order delta sigma method that is used to control the chemical resistance of MOX-based gas sensors has been introduced. This method improves one previously proposed by the authors, providing second-order quantization noise shaping, providing smoother responses, and allowing for the avoidance of the unwanted plateau-related phenomena that are typical of first-order strategies. The feasibility and the features of the new method have been demonstrated experimentally. Future work will be oriented towards optimizing controller parameters and architecture, improving the electronic readout of the sensor, and optimizing the sensor physical structure.
